# Supplementary figures and images for: Plasma pentosidine levels are associated with prevalent fractures in patients with chronic liver disease
Source: PLoS One. 2021 Apr 2;16(4):e0249728. doi: 10.1371/journal.pone.0249728 (PMC8018620; doi:10.1371/journal.pone.0249728)

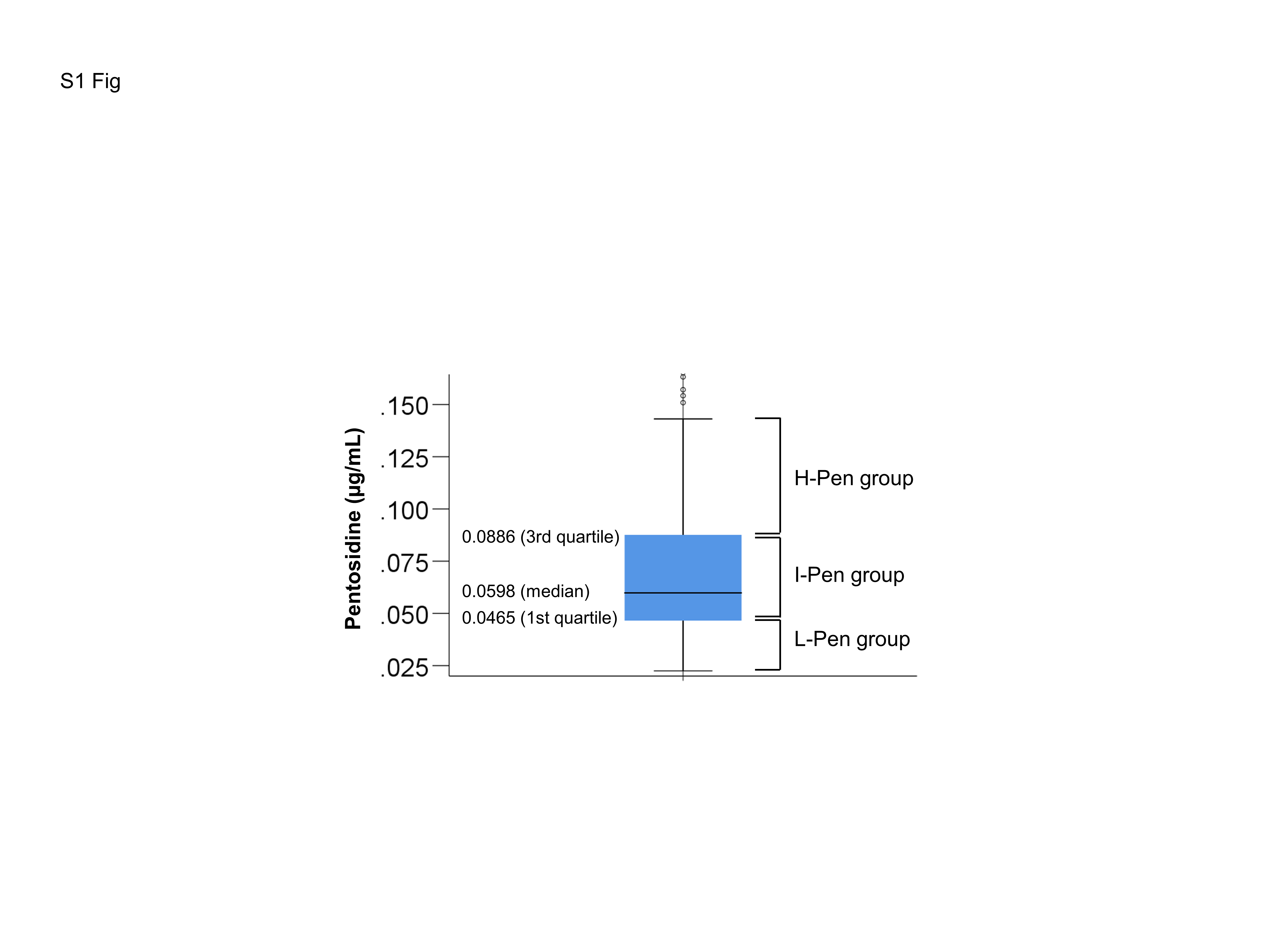

Supplement: S1 Fig — The median (interquartile range) pentosidine level was 0.0598 (0.0465–0.0886) μg/mL. The 324 patients were divided into three groups: (1) the low pentosidine (L-Pen) group had pentosidine levels ≤0.0465 μg/mL (first quartile); (2) the intermediate pentosidine group had pentosidine levels 0.0465–0.0886 μg/mL (third quartile); and (3) the high pentosidine group had pentosidine levels ≥0.0886 μg/mL. (TIF) [file pone.0249728.s001.tif]

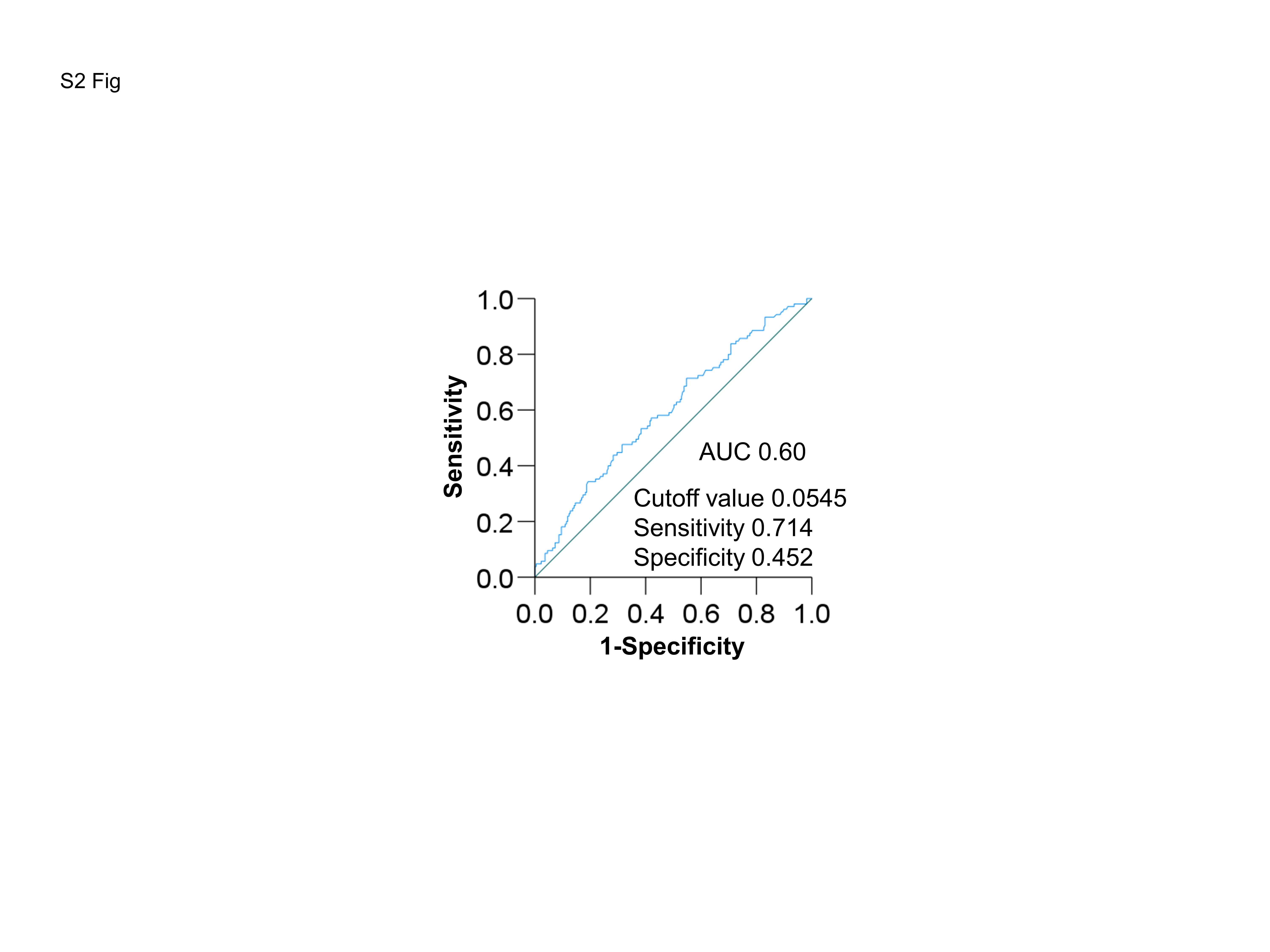

Supplement: S2 Fig — The plasma pentosidine cutoff value was 0.0545 μg/mL with area under the ROC curve (AUC), specificity, and sensitivity of 0.60, 0.714, and 0.452, respectively. (TIF) [file pone.0249728.s002.tif]
