# Supplementary material for: Plasma pentosidine levels are associated with prevalent fractures in patients with chronic liver disease
Source: PLoS One. 2021 Apr 2;16(4):e0249728. doi: 10.1371/journal.pone.0249728 (PMC8018620; doi:10.1371/journal.pone.0249728)
Supplement: S1 Table — (DOCX) [file pone.0249728.s003.docx]

**S1 Table. Comparison of baseline characteristics across etiologies**

| Variable | HBV | HCV | Alcohol | PBC | Others | *p* value |
| --- | --- | --- | --- | --- | --- | --- |
| Patients, n (%) | 46 (14.2) | 99 (30.6) | 63 (19.4) | 62 (19.1) | 54 (16.7) |  |
| Man, n (%) | 19 (41.3) | 61 (61.6) | 49 (77.8) | 12 (19.4) | 18 (33.3) | < 0.001 |
| Age (years) | 63.5 (53.8–71.3) | 75.0 (66.0–80.0) | 61.0 (52.0–72.0) | 68.5 (58.5–74.0) | 73.0 (59.0–78.0) | < 0.001 |
| BMI (kg/m^2^) | 23.3 (20.6–25.5) | 22.4 (20.3–24.7) | 23.1 (21.2–25.4) | 22.5 (20.3–25.6) | 25.9 (23.4–29.7) | < 0.001 |
| Current smoking | 6 (13.0) | 22 (22.2) | 39 (61.9) | 14 (22.6) | 5 (9.3) | < 0.001 |
| Diabetes mellitus, n (%) | 7 (15.2) | 20 (20.2) | 19 (30.2) | 13 (21.0) | 26 (48.1) | < 0.001 |
| Chronic kidney disease, (%) | 16 (34.8) | 45 (45.5) | 22 (34.9) | 28 (45.2) | 26 (48.1) | 0.428 |
| Liver cirrhosis, n (%) | 18 (39.1) | 58 (58.6) | 61 (96.8) | 11 (17.7) | 40 (74.1) | < 0.001 |
| Total bilirubin (mg/dL) | 0.8 (0.6–1.1) | 0.7 (0.5–0.9) | 1.0 (0.7–1.6) | 0.5 (0.4–0.8) | 0.8 (0.6–1.0) | < 0.001 |
| Albumin (g/dL) | 4.2 (3.9–4.4) | 4.1 (3.6–4.4) | 3.5 (3.1–3.9) | 4.0 (3.9–4.3) | 3.8 (3.5–4.1) | < 0.001 |
| Prothrombin time INR | 1.01 (0.97–1.10) | 1.06 (1.00–1.15) | 1.17 (1.07–1.33) | 0.98 (0.92–1.03) | 1.10 (1.00–1.17) | < 0.001 |
| Creatinine (mg/dL) | 0.8 (0.7–1.0) | 0.8 (0.7–1.1) | 0.8 (0.7–1.1) | 0.8 (0.6–0.9) | 0.8 (0.6–1.0) | 0.079 |
| eGFR (mL/min/1.73m^2^) | 66 (55–75) | 60 (50–72) | 72 (55–83) | 64 (52–77) | 63 (50–77) | 0.114 |
| M2BPGi (C.O.I) | 0.78 (0.60–1.43) | 2.16 (1.18–4.50) | 4.46 (2.06–7.15) | 1.12 (0.65–1.75) | 2.13 (1.31–4.10) | < 0.001 |
| IGF-1 (ng/mL) | 80 (52–107) | 60 (43–78) | 50 (34–67) | 72 (52–107) | 63 (46–82) | < 0.001 |
| 25(OH)D (ng/mL) | 15.2 (11.3–18.6) | 13.6 (10.5–17.1) | 11.1 (8.9–16.6) | 13.7 (10.4–18.3) | 12.9 (9.3–16.8) | 0.095 |
| Pentosidine (μg/mL) | 0.0543 (0.0401–0.0703) | 0.0635 (0.0486–0.0879) | 0.0773 (0.0558–0.1168) | 0.0549 (0.0449–0.0775) | 0.0554 (0.0444–0.0828) | < 0.001 |
| Lumbar spine BMD (g/cm^2^) | 1.02 (0.88–1.22) | 1.07 (0.91–1.21) | 1.14 (1.06–1.30) | 0.95 (0.84–1.17) | 1.04 (0.88–1.16) | 0.002 |
| Femoral neck BMD (g/cm^2^) | 0.74 (0.66–0.87) | 0.74 (0.64–0.87) | 0.84 (0.73–0.93) | 0.72 (0.65–0.87) | 0.77 (0.66–0.88) | 0.002 |
| Total hip BMD (g/cm^2^) | 0.81 (0.69–0.97) | 0.79 (0.69–0.92) | 0.89 (0.81–0.97) | 0.77 (0.67–0.89) | 0.83 (0.70–0.94) | 0.003 |
| Prevalent fracture, n (%) | 10 (21.7) | 35 (35.4) | 21 (33.3) | 18 (29.0) | 21 (38.9) | 0.384 |

Values are presented as medians (interquartile ranges) or relative frequencies (%). Statistical analysis was performed using the chi-squared test or the Kruskal-Wallis test, as appropriate. 25(OH)D, 25-hydroxyvitamin D; BMD, bone mineral density; BMI, body mass index; eGFR, estimated glomerular filtration rate; HBV, hepatitis B virus; HCV, hepatitis C virus; IGF-1, insulin-like growth factor 1; INR, international normalized ratio; M2BPGi, Mac-2 binding protein glycosylation isomer; PBC, primary biliary cholangitis.
