# Supplementary material for: Plasma pentosidine levels are associated with prevalent fractures in patients with chronic liver disease
Source: PLoS One. 2021 Apr 2;16(4):e0249728. doi: 10.1371/journal.pone.0249728 (PMC8018620; doi:10.1371/journal.pone.0249728)
Supplement: S2 Table — (DOCX) [file pone.0249728.s004.docx]

**S2 Table. Univariate analysis of factors associated with prevalent fractures**

| Variable | OR (95% CI) | *p* value |
| --- | --- | --- |
| Gender (Man) | 0.867(0.544–1.382) | 0.548 |
| Age (years) | 1.084(1.056–1.113) | < 0.001 |
| BMI (kg/m^2^) | 0.933(0.878–0.993) | 0.028 |
| Current smoking | 0.642(0.369–1.116) | 0.116 |
| Current drinking | 0.667(0.302–1.474) | 0.316 |
| Diabetes mellitus | 1.111(0.657–1.877) | 0.695 |
| Chronic kidney disease | 1.638(1.024–2.620) | 0.039 |
| Liver cirrhosis | 1.937(1.187–3.163) | 0.008 |
| Etiology | 1.101(0.922–1.314) | 0.287 |
| Total bilirubin (mg/dL) | 1.071(0.863–1.329) | 0.536 |
| Albumin (g/dL) | 0.753(0.510–1.112) | 0.715 |
| Prothrombin time INR | 1.300(0.319–5.300) | 0.715 |
| Creatinine (mg/dL) | 1.756(0.995–3.097) | 0.052 |
| eGFR (mL/min/1.73m^2^) | 0.982(0.969–0.994) | 0.005 |
| M2BPGi (C.O.I) | 1.036(0.964–1.114) | 0.335 |
| IGF-1 (ng/mL) | 0.984(0.976–0.993) | < 0.001 |
| 25(OH)D (ng/mL) | 0.995(0.957–1.034) | 0.805 |
| Vitamin D deficiency | 1.424(0.686–2.959) | 0.343 |
| Pentosidine (x10^2^) (μg/mL) | 1.038(1.007–1.068) | 0.014 |
| Lumbar spine BMD (g/cm^2^) | 0.071(0.022–0.232) | < 0.001 |
| Femoral neck BMD (g/cm^2^) | 0.002(0.000–0.014) | < 0.001 |
| Total hip BMD (g/cm^2^) | 0.002(0.000–0.013) | < 0.001 |
| Osteoporosis | 5.101(3.070–8.477) | < 0.001 |

25(OH)D, 25-hydroxyvitamin D; BMD, bone mineral density; BMI, body mass index; CI, confidence interval; eGFR, estimated glomerular filtration rate; IGF-1, insulin-like growth factor 1; INR, international normalized ratio; M2BPGi, Mac-2 binding protein glycosylation isomer; OR, odds ratio.
