# Supplementary material for: Plasma pentosidine levels are associated with prevalent fractures in patients with chronic liver disease
Source: PLoS One. 2021 Apr 2;16(4):e0249728. doi: 10.1371/journal.pone.0249728 (PMC8018620; doi:10.1371/journal.pone.0249728)
Supplement: S3 Table — (DOCX) [file pone.0249728.s005.docx]

**S3 Table. Correlation between plasma pentosidine levels and baseline characteristics**

| Variable | Correlation coefficient | | *p* value |
| --- | --- | --- | --- |
| Age (years) | 0.096 | 0.086 | |
| BMI (kg/m^2^) | -0.154 | 0.005 | |
| Total bilirubin (mg/dL) | 0.320 | < 0.001 | |
| Albumin (g/dL) | -0.445 | < 0.001 | |
| Prothrombin time INR | 0.449 | < 0.001 | |
| Creatinine (mg/dL) | 0.252 | < 0.001 | |
| eGFR (mL/min/1.73m^2^) | -0.171 | 0.002 | |
| M2BPGi (C.O.I) | 0.569 | < 0.001 | |
| IGF-1 (ng/mL) | -0.305 | < 0.001 | |
| 25(OH)D (ng/mL) | -0.183 | 0.001 | |
| Lumbar spine BMD (g/cm^2^) | -0.038 | 0.496 | |
| Femoral neck BMD (g/cm^2^) | -0.008 | 0.890 | |
| Total hip BMD (g/cm^2^) | -0.047 | 0.397 | |

25(OH)D, 25-hydroxyvitamin D; BMD, bone mineral density; BMI, body mass index; eGFR, estimated glomerular filtration rate; IGF-1, insulin-like growth factor 1; INR, international normalized ratio; M2BPGi, Mac-2 binding protein glycosylation isomer.
