# Supplementary material for: Plasma pentosidine levels are associated with prevalent fractures in patients with chronic liver disease
Source: PLoS One. 2021 Apr 2;16(4):e0249728. doi: 10.1371/journal.pone.0249728 (PMC8018620; doi:10.1371/journal.pone.0249728)
Supplement: S4 Table — (DOCX) [file pone.0249728.s006.docx]

**S4** **Table. Multiple regression analysis of factors associated with plasma pentosidine levels**

| Variable | Standardized coefficient β | *p* value |
| --- | --- | --- |
| BMI (kg/m^2^) | −0.083 | 0.015 |
| Total bilirubin (mg/dL) | 0.584 | < 0.001 |
| Albumin (g/dL) | −0.207 | < 0.001 |
| Prothrombin time INR | 0.143 | 0.001 |
| Creatinine (mg/dL) | 0.116 | 0.001 |
| Prevalent fracture | 0.083 | 0.015 |

BMI, body mass index; INR, international normalized ratio.
