# Supplementary material for: Plasma pentosidine levels are associated with prevalent fractures in patients with chronic liver disease
Source: PLoS One. 2021 Apr 2;16(4):e0249728. doi: 10.1371/journal.pone.0249728 (PMC8018620; doi:10.1371/journal.pone.0249728)
Supplement: S5 Table — (DOCX) [file pone.0249728.s007.docx]

**S5 Table. Baseline characteristics of patients with and without high pentosidine levels and/or osteoporosis**

| Variable | Osteoporosis (−) | Osteoporosis (−) | Osteoporosis (+) | Osteoporosis (+) | *p* value |
| --- | --- | --- | --- | --- | --- |
|  | High-Pen (−) | High-Pen (+) | High-Pen (−) | High-Pen (+) |  |
| Patients, n (%) | 170 (52.5) | 51 (15.7) | 75 (23.1) | 28 (8.6) |  |
| Man, n (%) | 93 (54.7) | 34 (66.7) | 21 (28.0) | 11 (39.3) | < 0.001 |
| Age (years) | 65.0 (56.8–73.0) | 63.0 (53.0–75.0) | 74.0 (68.0–80.0) | 75.5 (69.0–78.8) | < 0.001 |
| BMI (kg/m^2^) | 24.0 (21.9–26.8) | 22.4 (20.1–25.6) | 21.3 (20.2–24.2) | 22.0 (19.3–24.6) | < 0.001 |
| Current smoking, n (%) | 48 (28.4) | 18 (35.3) | 12 (16.0) | 8 (28.6) | 0.085 |
| Current drinking, n (%) | 15 (8.9) | 16 (31.4) | 3 (4.0) | 2 (7.1) | < 0.001 |
| Diabetes mellitus, n (%) | 48 (28.2) | 14 (27.5) | 15 (20.0) | 8 (28.6) | 0.577 |
| Chronic kidney disease, n (%) | 63 (37.1) | 23 (45.1) | 33 (44.0) | 18 (64.3) | 0.052 |
| Liver cirrhosis, n (%) | 80 (47.1) | 45 (88.2) | 38 (50.7) | 25 (89.3) | < 0.001 |
| Etiology |  |  |  |  |  |
| HBV/HCV/AL/PBC/other, n | 29/41/31/34/35 | 2/18/22/5/4 | 11/33/5/15/11 | 4/7/5/8/4 | < 0.001 |
| Total bilirubin (mg/dL) | 0.7 (0.5–0.9) | 1.3 (0.6–2.2) | 0.6 (0.5–0.8) | 0.8 (0.5–1.7) | < 0.001 |
| Albumin (g/dL) | 4.1 (3.8–4.4) | 3.3 (2.8–3.6) | 4.0 (3.8–4.4) | 3.6 (2.6–3.9) | < 0.001 |
| Prothrombin time INR | 1.03 (0.96–1.12) | 1.21 (1.08–1.39) | 1.03 (0.97–1.10) | 1.10 (1.03–1.22) | < 0.001 |
| Creatinine (mg/dL) | 0.8 (0.7–1.0) | 0.8 (0.7–1.1) | 0.7 (0.7–0.9) | 1.1 (1.0–1.2) | 0.019 |
| eGFR (mL/min/1.73m^2^) | 65 (54–77) | 65 (47–78) | 62 (53–73) | 52 (38–76) | 0.086 |
| M2BPGi (C.O.I) | 1.21 (0.70–2.54) | 6.69 (3.65–8.66) | 1.66 (1.18–2.83) | 3.61 (1.62–6.87) | < 0.001 |
| IGF-1 (ng/mL) | 76 (52–97) | 51 (36–64) | 60 (45–75) | 44 (27–55) | < 0.001 |
| 25(OH)D (ng/mL) | 14.2 (10.7–18.4) | 11.1 (9.0–15.0) | 13.7 (9.7–17.9) | 11.3 (8.1–14.8) | 0.010 |
| Pentosidine (μg/mL) | 0.0537 (0.0434–0.0647) | 0.1156 (0.1007–0.2247) | 0.0538 (0.0413–0.0669) | 0.1201 (0.9920–0.2182) | < 0.001 |
| Lumbar spine BMD (g/cm^2^) | 1.17 (1.03–1.27) | 1.09 (0.98–1.21) | 0.86 (0.76–0.99) | 0.87 (0.78–1.01) | < 0.001 |
| Femoral neck BMD (g/cm^2^) | 0.83 (0.75–0.91) | 0.84 (0.78–0.92) | 0.62 (0.56–0.67) | 0.61 (0.56–0.65) | < 0.001 |
| Total hip BMD (g/cm^2^) | 0.89 (0.82–0.97) | 0.89 (0.82–0.96) | 0.67 (0.61–0.71) | 0.65 (0.59–0.71) | < 0.001 |
| Prevalent fracture, n (%) | 32 (18.8) | 14 (27.5) | 38 (50.7) | 21 (75.0) | < 0.001 |

Values are presented as medians (interquartile ranges) or relative frequencies (%). Statistical analysis was performed using the chi-squared test or the Kruskal-Wallis test, as appropriate. 25(OH)D, 25-hydroxyvitamin D; AL, alcohol; BMD, bone mineral density; BMI, body mass index; eGFR, estimated glomerular filtration rate; HBV, hepatitis B virus; HCV, hepatitis C virus; IGF-1, insulin-like growth factor 1; INR, international normalized ratio; M2BPGi, Mac-2 binding protein glycosylation isomer; PBC, primary biliary cholangitis; Pen, pentosidine.
