# Supplementary material for: Plasma pentosidine levels are associated with prevalent fractures in patients with chronic liver disease
Source: PLoS One. 2021 Apr 2;16(4):e0249728. doi: 10.1371/journal.pone.0249728 (PMC8018620; doi:10.1371/journal.pone.0249728)
Supplement: Attachment — Submitted filename: Response to reviewers.docx [file pone.0249728.s009.docx]

**RESPONSES TO THE REVIEWER**

*We wish to express our deep appreciation to the reviewers for the constructive comments on our manuscript. Our point-by-point responses to the reviewers’ comments are listed below.*

**Review Comments to the Author:**

**Reviewer #1:** In their study the authors determined the relationship between plasma levels of pentosidine and prevalent fractures in 324 patients with chronic liver disease. As mentioned before, the presented data are of relevance, but nevertheless showed a couple of weaknesses in their first manuscript draft. In the revised version of their work, the authors responded to multiple comments. They included multivariable analyses taking possible confounding factors, such as the presence of CKD and diabetes mellitus into account and newly performed multiple regression analyses to identify significant and independent factors affecting plasma pentosidine levels.
Another major weakness of the study was represented by the great heterogeneity within the cohort regarding different liver disease etiologies. This issue still remains, but the authors now succeeded in improving the characterization of the study cohort and showed that liver disease etiology is not significantly associated with plasma pentosidine levels. However, the newly supplemented ROC curve analyses unfortunately suggested that plasma pentosidine levels may not be very useful in predicting fractures. The relationship between serum levels of pentosidine and prognosis in patients with liver disease should be investigated in the future.
Overall, the authors have now managed to pay more attention to confounding factors and to better characterise the cohort.

**Responses:** We are deeply grateful for the reviewer’s encouraging comments. We feel the previous reviewers’ comments have helped us markedly improve our manuscript. In the future, we are willing to investigate and report the relationship between plasma pentosidine levels and prognosis in patients with CLD.

**Reviewer #2:** Dear Authors, thank you for providing modifications in the revised protocol. Almost all major and minor concerns have been addressed.
An additional minor revision would raise the overall value:
1. The authors have performed AUROC analysis for the optimal pentosidine cut-off value for predicting prevalent fractures (R2). Please feel encouraged to include and disclose this data in the final submission (at the moment only available for review) in an effort to increase transparency. Additionally, an open discussion on the implications issued by the underlying data is desirable.

**Responses:** As suggested by the reviewer, we included the results of AUROC analysis in the final manuscript (S2 Figure). As shown in the first revise, the plasma pentosidine cutoff value for predicting prevalent fractures, AUC, sensitivity, and specificity were 0.0545 μg/mL, 0.60, 0.714, and 0.452, respectively. These results suggest that the plasma pentosidine levels are not very useful for predicting prevalent fractures in our cross-sectional study. In the furture, a large-scale multicenter, prospective studies are needed to conclude the usefulness of plasma pentosidine in predicting the occurrence of further fragility fractures.
